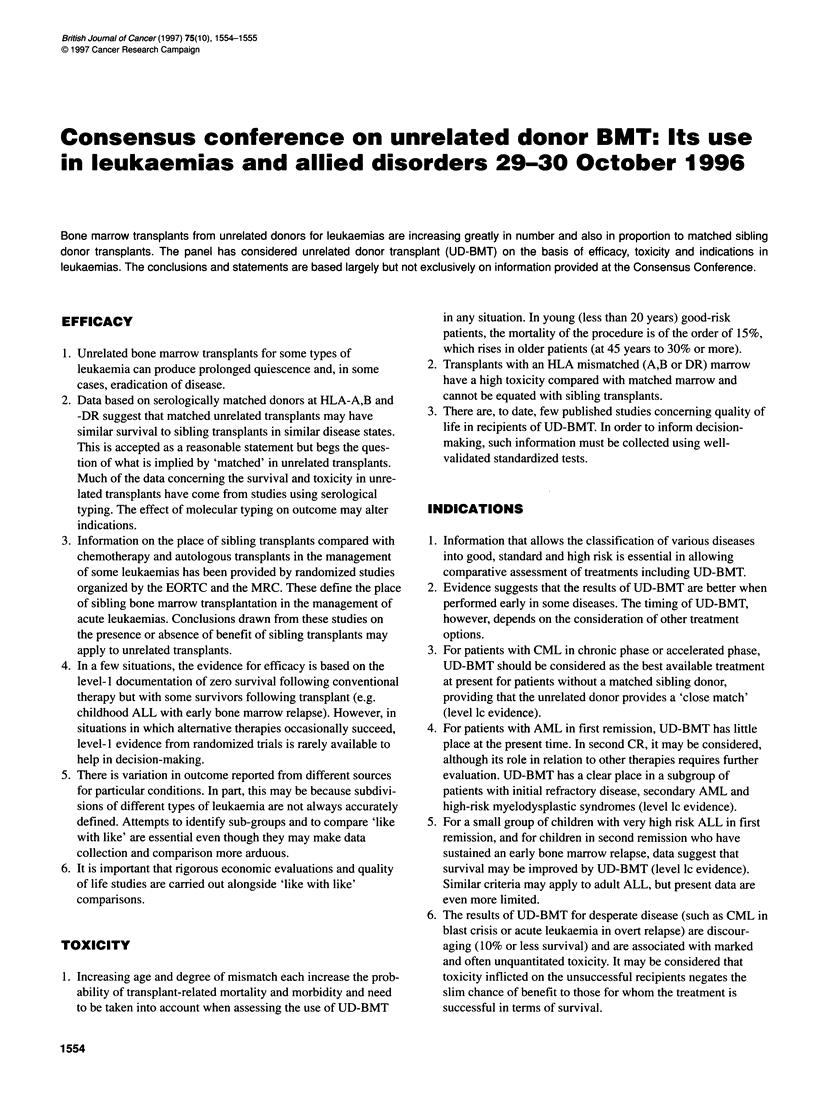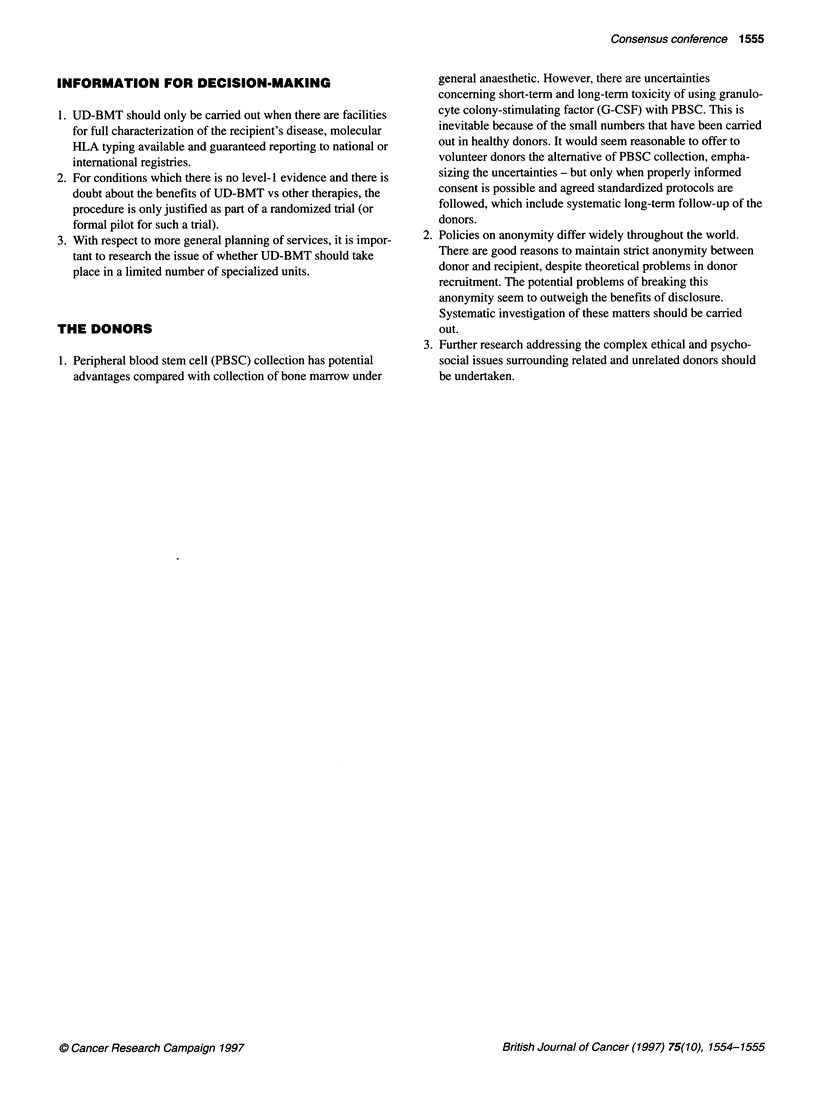# Consensus conference on unrelated donor BMT: Its use in leukaemias and allied disorders 29-30 October 1996

**Published:** 1997

**Authors:** 


British Journal of Cancer (1997) 75(10), 1554–1555
© 1997 Cancer Research Campaign

### Consensus conference on unrelated donor BMT: Its use in leukaemias and allied disorders 29–30 October 1996

Bone marrow transplants from unrelated donors for leukaemias are increasing greatly in number and also in proportion to matched sibling donor transplants. The panel has considered unrelated donor transplant (UD-BMT) on the basis of efficacy, toxicity and indications in leukaemias. The conclusions and statements are based largely but not exclusively on information provided at the Consensus Conference.

### EFFICACY

1. Unrelated bone marrow transplants for some types of leukaemia can produce prolonged quiescence and, in some cases, eradication of disease.
2. Data based on serologically matched donors at HLA-A,B and -DR suggest that matched unrelated transplants may have similar survival to sibling transplants in similar disease states. This is accepted as a reasonable statement but begs the question of what is implied by 'matched' in unrelated transplants. Much of the data concerning the survival and toxicity in unrelated transplants have come from studies using serological typing. The effect of molecular typing on outcome may alter indications.
3. Information on the place of sibling transplants compared with chemotherapy and autologous transplants in the management of some leukaemias has been provided by randomized studies organized by the EORTC and the MRC. These define the place of sibling bone marrow transplantation in the management of acute leukaemias. Conclusions drawn from these studies on the presence or absence of benefit of sibling transplants may apply to unrelated transplants.
4. In a few situations, the evidence for efficacy is based on the level-1 documentation of zero survival following conventional therapy but with some survivors following transplant (e.g. childhood ALL with early bone marrow relapse). However, in situations in which alternative therapies occasionally succeed, level-1 evidence from randomized trials is rarely available to help in decision-making.
5. There is variation in outcome reported from different sources for particular conditions. In part, this may be because subdivisions of different types of leukaemia are not always accurately defined. Attempts to identify sub-groups and to compare 'like with like' are essential even though they may make data collection and comparison more arduous.
6. It is important that rigorous economic evaluations and quality of life studies are carried out alongside 'like with like' comparisons.

### TOXICITY

1. Increasing age and degree of mismatch each increase the probability of transplant-related mortality and morbidity and need to be taken into account when assessing the use of UD-BMT in any situation. In young (less than 20 years) good-risk patients, the mortality of the procedure is of the order of 15%, which rises in older patients (at 45 years to 30% or more).
2. Transplants with an HLA mismatched (A,B or DR) marrow have a high toxicity compared with matched marrow and cannot be equated with sibling transplants.
3. There are, to date, few published studies concerning quality of life in recipients of UD-BMT. In order to inform decision-making, such information must be collected using well-validated standardized tests.

### INDICATIONS

1. Information that allows the classification of various diseases into good, standard and high risk is essential in allowing comparative assessment of treatments including UD-BMT.
2. Evidence suggests that the results of UD-BMT are better when performed early in some diseases. The timing of UD-BMT, however, depends on the consideration of other treatment options.
3. For patients with CML in chronic phase or accelerated phase, UD-BMT should be considered as the best available treatment at present for patients without a matched sibling donor, providing that the unrelated donor provides a 'close match' (level lc evidence).
4. For patients with AML in first remission, UD-BMT has little place at the present time. In second CR, it may be considered, although its role in relation to other therapies requires further evaluation. UD-BMT has a clear place in a subgroup of patients with initial refractory disease, secondary AML and high-risk myelodysplastic syndromes (level lc evidence).
5. For a small group of children with very high risk ALL in first remission, and for children in second remission who have sustained an early bone marrow relapse, data suggest that survival may be improved by UD-BMT (level lc evidence). Similar criteria may apply to adult ALL, but present data are even more limited.
6. The results of UD-BMT for desperate disease (such as CML in blast crisis or acute leukaemia in overt relapse) are discouraging (10% or less survival) and are associated with marked and often unquantitated toxicity. It may be considered that toxicity inflicted on the unsuccessful recipients negates the slim chance of benefit to those for whom the treatment is successful in terms of survival.

### INFORMATION FOR DECISION-MAKING

1. UD-BMT should only be carried out when there are facilities for full characterization of the recipient's disease, molecular HLA typing available and guaranteed reporting to national or international registries.
2. For conditions which there is no level-1 evidence and there is doubt about the benefits of UD-BMT vs other therapies, the procedure is only justified as part of a randomized trial (or formal pilot for such a trial).
3. With respect to more general planning of services, it is important to research the issue of whether UD-BMT should take place in a limited number of specialized units.

### THE DONORS

1. Peripheral blood stem cell (PBSC) collection has potential advantages compared with collection of bone marrow under general anaesthetic. However, there are uncertainties concerning short-term and long-term toxicity of using granulocyte colony-stimulating factor (G-CSF) with PBSC. This is inevitable because of the small numbers that have been carried out in healthy donors. It would seem reasonable to offer to volunteer donors the alternative of PBSC collection, emphasizing the uncertainties – but only when properly informed consent is possible and agreed standardized protocols are followed, which include systematic long-term follow-up of the donors.
2. Policies on anonymity differ widely throughout the world. There are good reasons to maintain strict anonymity between donor and recipient, despite theoretical problems in donor recruitment. The potential problems of breaking this anonymity seem to outweigh the benefits of disclosure. Systematic investigation of these matters should be carried out.
3. Further research addressing the complex ethical and psychosocial issues surrounding related and unrelated donors should be undertaken.

© Cancer Research Campaign 1997